# A Novel Sample Based Quadrature Phase Shift Keying Demodulator

**DOI:** 10.1155/2014/107831

**Published:** 2014-08-14

**Authors:** Asraf Mohamed Moubark, Sawal Hamid Md Ali

**Affiliations:** Department of Electrical, Electronic and Systems Engineering, Universiti Kebangsaan Malaysia, 43600 Bangi, Selangor, Malaysia

## Abstract

This paper presents a new practical QPSK receiver that uses digitized samples of incoming QPSK analog signal to determine the phase of the QPSK symbol. The proposed technique is more robust to phase noise and consumes up to 89.6% less power for signal detection in demodulation operation. On the contrary, the conventional QPSK demodulation process where it uses coherent detection technique requires the exact incoming signal frequency; thus, any variation in the frequency of the local oscillator or incoming signal will cause phase noise. A software simulation of the proposed design was successfully carried out using MATLAB Simulink software platform. In the conventional system, at least 10 dB signal to noise ratio (SNR) is required to achieve the bit error rate (BER) of 10^−6^, whereas, in the proposed technique, the same BER value can be achieved with only 5 dB SNR. Since some of the power consuming elements such as voltage control oscillator (VCO), mixer, and low pass filter (LPF) are no longer needed, the proposed QPSK demodulator will consume almost 68.8% to 99.6% less operational power compared to conventional QPSK demodulator.

## 1. Introduction

Quadrature Phase Shift Keying (QPSK) is a modulation scheme commonly used in wireless communication system due to its ability to transmit twice the data rate for a given bandwidth [1]. An ideal QPSK signal where the in-phase and quadrature components are in quadrature (90°) and have equal amplitude cannot be obtained, according to [2] due to the noise presence in local oscillator, DC offset in mixer, and phase imbalance in power combiner and mixers. At the same time, the transmitted QPSK signal frequency, *ω*_*c*_, may also vary by Δ*ω* due to Doppler effect [3]. A practical QPSK signal, *s*(*t*), will contain gain error (*ε*), phase error (*β*), and frequency shift (Δ*ω*), as given in

(1) $$\begin{array}{c} s\left(t\right)=\left(1+\varepsilon \right)\sqrt{\frac{2\text{Es}}{T}}\cos\left(\left(\omega_{c}+\Delta \omega \right)t+\theta +\beta \right), \end{array}$$

where fc is the carrier frequency, Es is the energy per symbol, *T* is the symbol period, and *θ* is the carrier phase. While the gain error can be easily reduced with automatic gain controller (AGC), the phase noise and frequency shift are more complicated and can tremendously affect the performance of the system. The performance of a conventional coherent demodulator starts to degrade at phase noise of 3.6° [4]. Previous works attempted to solve both phase error and frequency shift problems by using feedback control loop and feed-forward compensation technique, only adding more complexity to the demodulator circuit [5].

Typical QPSK demodulator needs SNR of 10 dB to produce BER of 10^−6^ [1]. Even though the SNR value presumed as low in wireless signal transmission, for a system such as satellite and mobile devices where their operations are power limited, this is an issue that needs attention [6]. Components used in the conventional QPSK demodulator for the demodulation process such as VCO, LPF, and mixers increase the power consumption of the system. Thus, this paper proposed a new technique that is simple, consumes less power, and is robust to the phase noise.

This paper is structured as follows.  Section 1 gives an introduction to readers about this journal.  Section 2 briefly explains the proposed new QPSK demodulator, followed by Section 3 showing MATLAB simulation carried out on the proposed architecture. In Section 4 results obtained from the simulation are provided and discussed in detail. Finally our results are concluded and some details about our future work are given in Section 5.

## 2. Proposed QPSK Demodulator

**Figure 1:**
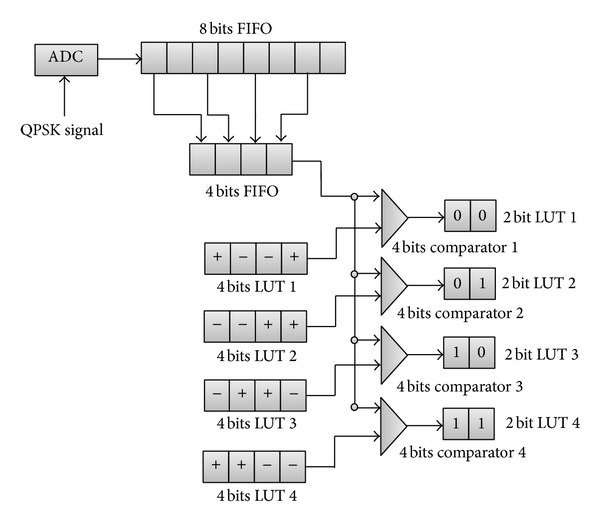
Block diagram for the proposed 8S-QPSK demodulator.

The proposed QPSK demodulator uses polarity difference from digitized QPSK signal for the demodulation process and is given a new code name 8S-QPSK.  Figure 1 shows the complete block diagram for the proposed design where it consists of analog to digital converter (ADC), first in first out (FIFO), lookup table (LUT), and comparators.

Digitizing is a process used in ADC to convert the incoming analog signal to digital signal based on the ADC sampling rate. In the proposed QPSK demodulator the incoming signal is sampled 8 times of the incoming signal frequency. A sample is produced for every rising clock of the ADC circuit for a total number of 8 samples. A decision is made on every 2 samples to be classified as positive and negative samples based on the sample's polarities. This will eventually produce 4 different polarities for a QPSK signal and they are different for every QPSK symbol as shown in Table 1.

The QPSK phases represent a group of 2 bits data. The phases are produced at modulation level according to the inputs bits to the sinusoidal carrier [7, 8]. Once the phases are identified, the data can be recovered immediately.

## 3. MATLAB Simulation 

The whole demodulation process starts with sampling of the QPSK signal from the signal recovery block by using the sample and hold circuit block. The sample and hold block will convert the continuous QPSK signal, *s*(*t*), into discrete signal, *S*[*n*], as given by

(2) $$\begin{array}{c} S\left[n\right]=\frac{s\left(t\right)}{V_{r}}\times 2^{y}, \end{array}$$

where *n* is the number of samples and *y* is the quantization level. The maximum range of the ADC voltage is ±*V*_*r*_ centered on the reference voltage 0 v. The chosen transmitted carrier frequency was 5 MHz because it is a frequently used bandwidth in wireless systems [9]. Therefore the sampling clock frequency used in sample and hold block was set to 8 times the incoming frequency which is 40 MHz. The sampling clock is set by using pulse generator where the period of the pulse can be programmed. A group, *a*_*k*_{  }, of 8 samples, *S*[*n*], are produced for every phase of a QPSK symbol as given by

(3) $$\begin{array}{c} a_{k}\left\{\;\right\}=S\left[n\right],\;k=1\cdots \infty ,\;\;n=1\sim 8. \end{array}$$

Continuously, from the odd samples, *S*[2*m* + 1] of *a*_*k*_{  }, a decision is made and sorted according to their polarities, *b*_*k*_{  }, as given by

(4) $$\begin{array}{c} b_{l}\left\{\;\right\}=\{\begin{array}{cc} 1, & \;S\left[2m+1\right]\;>0\\ -1, & S\left[2m+1\right]\;<0 \end{array}\\ m=0,1,2,3,\;m\in n,\;l=1\cdots \infty . \end{array}$$

**Figure 2:**
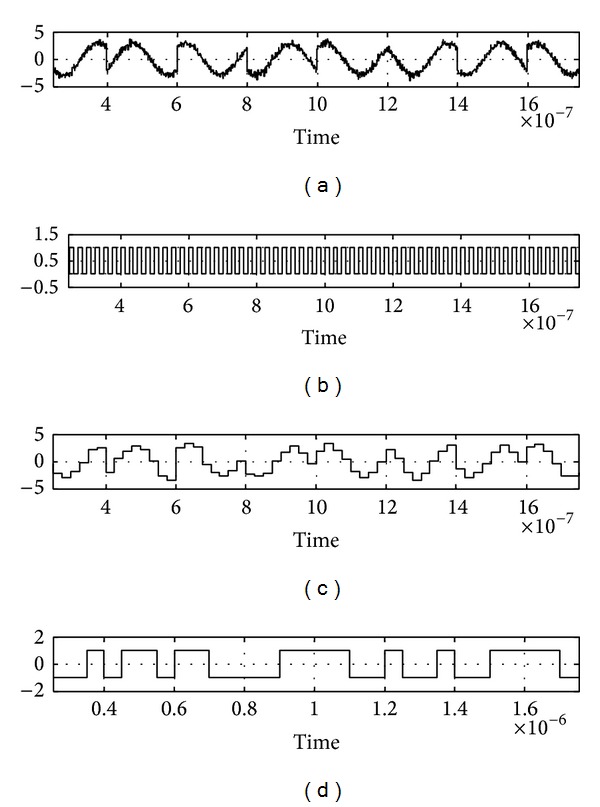
(a) The QPSK signal, (b) sampling time, (c) output from sampled QPSK signal, and (d) four samples obtained from the sampled QPSK signal.

The sign block is used after the sampling process to rearrange the sampled data according to the polarity.  Figure 2 shows in detail the sampling and grouping process.

### 3.1. Buffer

The series of polarities samples need to be changed into a parallel of 4 × 1 matrixes so that it can be compared with each of the group data stored inside the LUT. A buffer with output size of four elements is used to redistribute the pulses from the sign block. Each pulse with positive and negative polarity has *t*_*s*_ period and needs to be grouped into four elements with period of *T* as given by

(5) $$\begin{array}{c} {T=4t}_{s},\;s=1\cdots \infty . \end{array}$$

**Figure 3:**
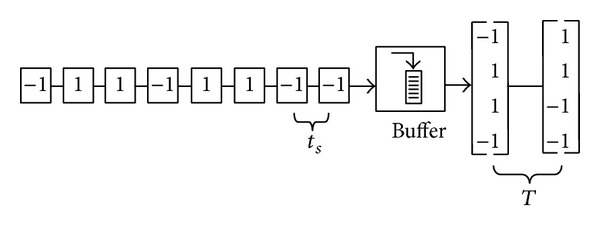
Buffer redistributes the incoming samples into 2 sets of samples.

This is crucial since only four complete pulses which have period equal to *T* can determine a combinational group of data represented by the QPSK signal.  Figure 3 shows an example on how the buffer redistributes the received data.

### 3.2. Lookup Table

A total number of four lookup tables (LUT) are used to store the 4 combinational pulses which were predetermined earlier. The data inside each LUT is stored in the form of 4 × 1 matrixes as shown in Figure 1. To do so, arrays of four constants data are grouped and transposed. Every LUT will be compared constantly with the buffer output which is also in the 4 × 1 matrix. The sample time inside the LUT was set at *T* so that one LUT can be compared with four pulses which have the same period. The LUT was not stacked in any order since the modulated data was in random order.

### 3.3. Comparator

A comparator is used to compare the data stored inside the LUT with the data from the buffer. If a group of four datasets from the buffer match with any LUT, this means that the QPSK symbol was sampled correctly and the dibits are able to be retrieved. Four embedded MATLAB function blocks are used for the comparator which contains a MATLAB function of *y* = isequal(*u*, *v*), where the *u* and *v* represent the data from the buffer and LUT, respectively.

## 4. Results and Discussions 

A MATLAB Simulink simulation was carried out on both of the QPSK demodulation techniques and compared to identify their performances with and without phase error. BER has been used as a main performance indicator in this project. The satisfactory BER values obtained for every different noise level in Simulink simulation are compiled in tables and represented in graphical forms. A total amount of 20 Mb of data was used as input to the modulator for every case in obtaining the BER values. At the same time, a study was carried out to evaluate the proposed design operating power consumption.

### 4.1. Performance Analysis on Signal Power with AWGN

**Figure 4:**
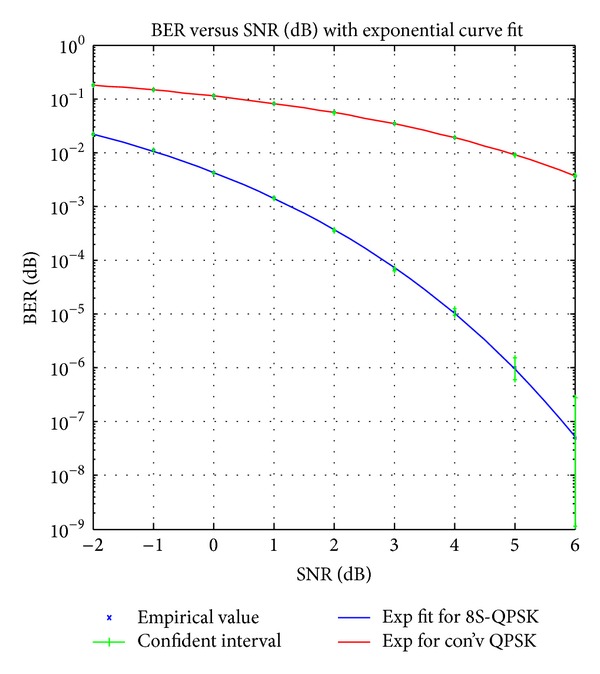
Performance comparison of 8S-QPSK and conventional QPSK scheme in additive white Gaussian noise (AWGN) channel with confidence interval and empirical value.

In this section, the QPSK signal is demodulated with the proposed and conventional demodulator with different levels of SNR starting from −2 dB until as high as 6 dB through AWGN channel. This analysis demonstrates the ability of the proposed architecture to withstand the white Gaussian noise compared to the conventional architecture. To validate the performance of the system a 95% of confidence level of the confidence interval test was used for every simulation. The confidence interval test was carried out by using MATLAB built-in function where it requires data such as BER, the total number of input data, and range of SNR to calculate the interval level and the maximum and minimum number of BER [10, 11]. The data compiled for both demodulation schemes are represented graphically in Figure 4. The exponential curve fit has been used to interpolate the log-log scale dataset so that the graph will resemble the water fall curve shape [12].

It can be seen from Figure 4, for a particular BER (e.g., 10^−2^), that the proposed technique has a lower SNR compared to the conventional technique. The noise power, *P*_noise_, for both signals can be determined using (6) and this noise power will be used together with the SNR value to give the signal power, *P*_signal_, as depicted in (7).  Table 2 shows the power comparison for both techniques at BER of 10^−2^:

(6) $$\begin{array}{c} P_{\text{noise}}=\left(-173.83+{10\log}_{10}B\right)\text{dB}m \end{array}$$

(7) $$\begin{array}{c} {\text{SNR}}_{\text{dB}}=10\;\log_{10}\frac{P_{\text{signal}}}{P_{\text{noise}}}. \end{array}$$

The result shows that, for a particular BER, the proposed method can reduce the signal detection power up to 74.9% when compared with conventional method and lower SNR values are obtained for every BER value. This is due to the architecture of the proposed design which no longer employs coherent detection technique in the demodulation process.

### 4.2. Performance Analysis on Signal Power with Phase Error

**Figure 5:**
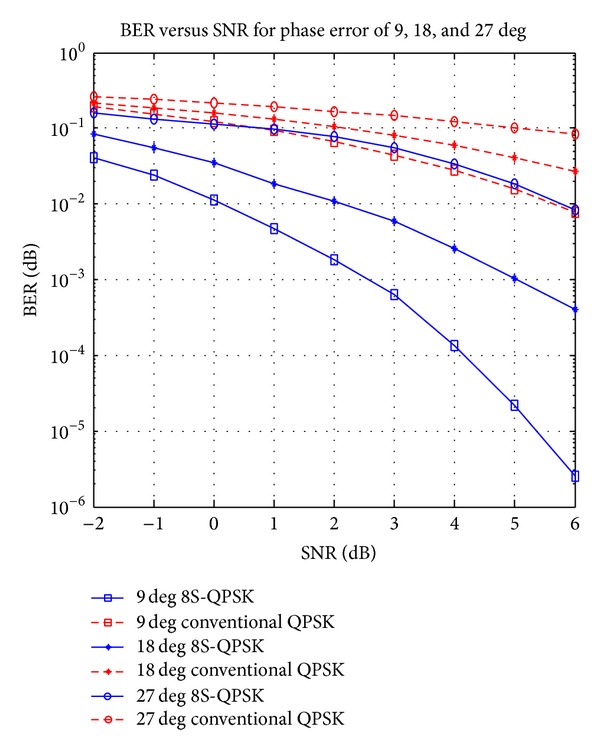
Performance comparison of 8S-QPSK and conventional QPSK signal with phase errors of 9°, 18°, and 27°.

**Figure 6:**
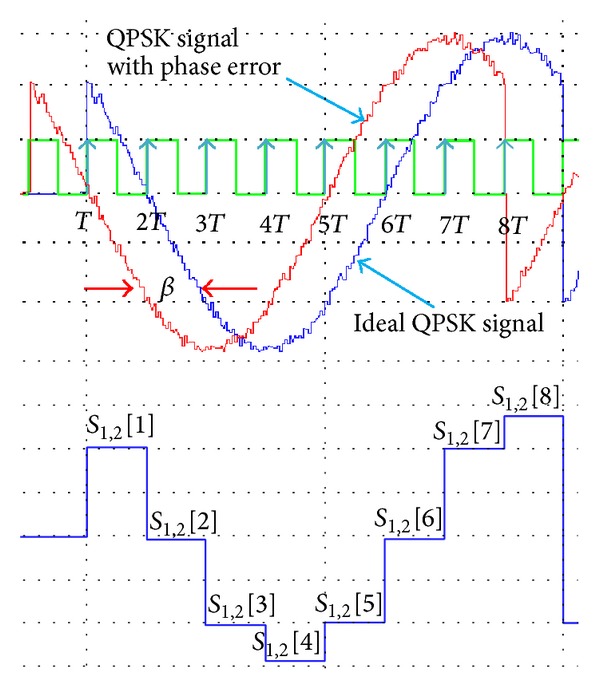
Samples obtained for phase error and standard QPSK signals from *n* = 1 until *n* = 8.

To determine the ability of the proposed design to tolerate high phase error in AWGN channel, a simulation has been performed to obtained data for BER with respect to SNR for 8S-QPSK and conventional QPSK schemes with phase errors of 9°, 18°, and 27°. The results obtained are shown in graphical form in Figure 5. SNR values for BER of 10^−2^ are taken from each graph and shown in Table 3 for comparison. As for phase error of 18° and 27° for conventional QPSK schemes, the SNR values are only shown until 6 dB in Figure 6. However, the other two SNR values, 8 and 11.6 dB, have been successfully obtained in the simulation conducted. In this simulation, confidence interval test is not included since the upper and lower limit values tend to overlap with each other between the curves.

The proposed demodulator shows power gain of 5.2 dB for 9° and 5.8 dB for 18° and 27° phase errors when compared to conventional QPSK demodulator. This achievement is obtained because the samples used to identify the signal phase, *θ*, are taken for every 45 degrees. Thus, if any variation happened on the signal between the sampling period, it is not going to affect the sample value obtained. As in Figure 6, the samples, *S*_1,2_[*n*], represent ideal QPSK signal, *S*_1_[*n*], and phase error QPSK signal by 45 degrees, *S*_2_[*n*]. As for both signals, samples obtained at any sampling time, *nT*, give the same positive or negative values regarding the phase error. The signals shown in Figure 6 are QPSK signals for *θ*, 45° generated at high SNR value, and 10 dB to give an idea on how the phase error alone will affect the sampling process:

(8) S1[n]=S2[n]=2m2(cos⁡⁡(ωcnT+θ+β1,2))Vr∈{±1},β1=0°, β2=45°.

However, when SNR values for 8S-QPSK from Tables 3 and 2 are compared, it shows that there are power increments from 1.2 dB to 6.8 dB to obtain the same BER value. The degradation on the performance happened due to the dependency of the proposed design on the amplitude of the incoming signal. The white Gaussian noise will cause voltage fluctuation on the incoming signal and thus will cause the polarity change in the sampling process. In conventional method, the phase error with the presents of white noise cannot be corrected or rectified with high SNR value as in proposed method.

To demonstrate the phase error effect on conventional demodulator, gain error and frequency shift have been removed from the incoming QPSK signal as in (1) to ease the calculation. At the same time, the phase error only was included into one of two local oscillators, sine carrier. This is to show clearly how phase error on one of the carriers can cause the demodulated data to be shifted in time domain when odd and even data were data merged together.

**Figure 7:**
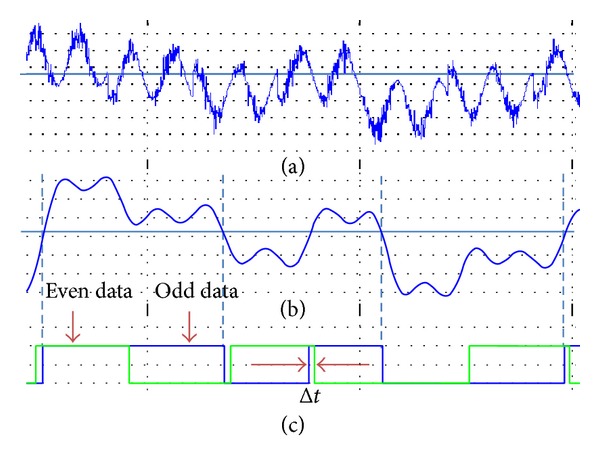
(a) Output from mixer of QPSK and sine carrier, (b) filtered I-channel signal with LPF, and (c) showing the mismatch in time domain between even and odd data due to phase error in sine carrier.

It can be seen that, by mixing the QPSK signal with the sine carrier as shown in (9), it produces two different terms. First, a sine signal with twice the frequency, phase shifted and half amplitude from the incoming signal. Second, another sine signal which varies according to the phase error and half the input amplitude signal. The first signal will be superimposed on second signal which acts like dc offset signal. By using an LPF after the mixing process, the first high frequency term, 2*ω*_*c*_, can be filtered out and the remaining term given in the expression will cause the odd data to be shifted in time domain by Δ*t*.  Figure 7 shows output from mixing the QPSK and sine carrier, filtered I-channel signal with LPF, and odd and even data obtained from I channel and Q-channel. The steps involved in obtaining the even data are not shown here but they are the same as in (9). The only difference is the sine carrier substituted by cosine carrier without any phase error:

(9) 2EsTcos⁡⁡(ωct+θ)×2 sin⁡(ωct+β) =2EsT[ej(ωct+θ)+e−j(ωct+θ)2×ej(ωct+β)−e−j(ωct+β)2j] =4EsT[  sin⁡(2ωct+θ+β)2−  sin⁡(θ−β)2].

It has been proven that a small variation (3.6°) of phase error will seriously affect the demodulation process and cause the data to be misinterpreted. On the other hand, the new architecture uses polarity of samples to recognize the QPSK signal symbols correctly and is not bounded with mixing signal issue mentioned earlier.

### 4.3. Analysis of Power Consumption

Power consumption estimation has been conducted for the proposed design based on the power consumed by individual components obtained from literature as shown in Table 4. For each one of the components, maximum and a minimum values are taken into consideration from various references so that it will give a rough idea for the total power consumption that can be expected.

The number of components used in the simulation is as follows: 1 unit of ADC, 16 units of 4 × 4 comparator, 8 units of LUT, and 2 units of FIFO as shown in Figure 1. The following calculation shows the maximum and minimum power consumption for the proposed design:

(10) P8-QPSK(Min⁡) =1×PADC+16×Pcomparator+8×PLUT+2×PFIFO =0.732 mW+16(3.2 uW)+8(0.274 uW)+2(1.39 uW) =0.788 mWP8-QPSK(Max⁡) =1×PADC+16×Pcomparator+8×PLUT+2×PFIFO =62 mW+16(36.25 uW)+8(26.452 uW)+2(721 uW) =64.233 mW.

It can be seen that the power consumption of the proposed design is between 0.788 mW and 64.233 mW which is significantly reduced compared to the 206 mW [20] consumed by the conventional QPSK. The reduction of the power consumption is between 68.8 and 99.6%.

## 5. Conclusion and Future Work

A novel architecture for QPSK demodulator has been proposed and demonstrated promising results. The results obtained for both demodulation schemes do not include any error correction coding, phase, and frequency error detection technique. The new demodulation technique uses samples polarity from ADC to identify the QPSK symbols. This indirectly eliminates the use of VCO, a component that contributes to the phase and frequency distortion. The new architecture consumes almost 74.9% less power for signal detection in AWGN channel without any phase error and 50 to 89.6% when phase error is presented. On top of that, the proposed design is also expected to consume 68.8 to 99.6% less power and significant size reduction compared with conventional architecture.

As for future work, channel selection method, error correction coding, and signal locking mechanism for sampling starting time will be included to further evaluate the proposed architecture. Hardware implementation on Virtex 6 FPGA board has been planned for performance measurement and verification.

## Figures and Tables

**Table 1:** Combinational polarities for QPSK symbols.

Phase	Data	4 different polarities			
45°	00	**+**	**−**	**−**	**+**
135°	01	**−**	**−**	**+**	**+**
225°	10	**−**	**+**	**+**	**−**
315°	11	**+**	**+**	**−**	**−**

**Table 2:** Comparison of signal power between the two schemes.

Demodulation scheme	SNR for 10*E* − 2 (dB)	Noise power for 6.75 MHz (W)	Signal power (W)	% of power saved
Conventional QPSK	5	2.78*E* − 14	8.79*E* − 14	0
8S-QPSK	−1	2.78*E* − 14	2.20*E* − 14	74.9%

**Table 3:** Comparison of power gain between the 3 phases error at BER of 10^−2^.

Phase error	Conventional QPSK (dB)	8S-QPSK (dB)	Power gain (dB)
9°	5.8	0.2	5.2
18°	8	2.2	5.8
27°	11.6	5.8	5.8

**Table 4:** Power comparison for various components in QPSK demodulator.

Components	Researcher	Power	Speed
ADC	[13]	0.732 mW	1 GSps
	[14]	62 mW	1.4 GSps

Comparator	[15]	3.2 uW	—
	[13]	36.25 uW	—

LUT	[16]	0.274 uW	2.98 GHz
	[17]	26.452 uW	—

FIFO	[18]	1.39 uW	—
	[19]	721 uW	2 GHz
